# Exact group sequential designs for two-arm experiments with Poisson distributed outcome variables

**DOI:** 10.1080/03610926.2019.1628273

**Published:** 2019-06-17

**Authors:** Michael J. Grayling, James M. S. Wason, Adrian P. Mander

**Affiliations:** a MRC Biostatistics Unit, Hub for Trials Methodology Research, Cambridge, UK; b Institute of Health & Society, Newcastle University, Newcastle, UK; c Centre for Trials Research, Cardiff University, Cardiff, UK

**Keywords:** Adaptive design, exact, interim analysis, optimal, Skellam, two-stage

## Abstract

We describe and compare two methods for the group sequential design of two-arm experiments with Poisson distributed data, which are based on a normal approximation and exact calculations respectively. A framework to determine near-optimal stopping boundaries is also presented. Using this framework, for a considered example, we demonstrate that a group sequential design could reduce the expected sample size under the null hypothesis by as much as 44% compared to a fixed sample approach. We conclude with a discussion of the advantages and disadvantages of the two presented procedures.

## 1 Introduction

It is desirable that experiments be designed and analyzed to ensure control of their type-I and type-II error-rates. For, within the context of a clinical trial, for example, an inflated type-I error-rate reduces our confidence that a significant health benefit has not been observed by chance, whilst under-powering a study raises the risk of failing to identify an efficacious treatment. It is for this reason that much research has been conducted to develop methodology for type-I error-rate control and sample size determination, in studies with many possible types of data, and many possible choices of null hypothesis. Within the context of experiments with discrete outcome variables, this research has often focused on the establishment of methods that are able to determine operating characteristics exactly. This allows us to reduce our reliance on approximate techniques that typically depend on asymptotic results. See, for example, Jung (2012), which summarizes exact methods for experiments with binary outcomes.

In contrast, for randomized two-arm experiments with Poisson distributed outcome variables, several authors have proposed approximate procedures for study design (Thode 1997; Shiue and Bain 1982; Mathews 2010), but it was only comparatively recently that Menon et al. (2011) described an alternative approach based on the exact distribution of the difference between two Poisson variables. They demonstrated that when the anticipated difference between the Poisson rates in the two arms was large, the sample size required by their approach was typically 5-10% smaller than that based on a normal approximation.

This result is a valuable one, as count outcomes occur in many experimental design settings of practical interest. In particular, within the context of clinical research, they are common in the form of the number of observed adverse events. More specifically, count outcomes occur as an endpoint of interest in epilepsy studies through seizure counts, in multiple sclerosis or Parkinson’s trials via relapse counts, and in migraine treatment studies as the number of attacks. So to are they useful in cardiovascular trials as the number of hospitalizations observed over a particular time-frame, or in alcohol treatment studies as the number of drinks over a recent period of time. Whilst in asthma trials, the number of exacerbations is often of interest, and as we will discuss later, the number of apnea and hypopnea events per hour is a common primary outcome in sleep-apnea studies.

Regardless of the now available efficient exact method noted above though, it is always of interest to be able to develop methods for reducing requisite sample sizes further. One widely applicable approach to achieving this is to employ a group sequential design, through which an experiment’s expected sample size (ESS) can be reduced by permitting early stopping following the repeated testing of a hypothesis of interest. Depending on the context, this can provide valuable savings in terms of time or money. For further details on group sequential methods see Jennison and Turnbull (2000).

Unfortunately, compared to settings with normally or Bernoulli distributed data, there is relatively little methodology available pertaining to the group sequential design of trials with Poisson distributed outcome variables. In fact, this is true more generally of count outcomes. Notable exceptions include the work of Cook and Lawless (1996), who described a non-parametric approach to interim monitoring in comparative studies with recurrent event outcomes. Moreover, Jiang (1999) considered the group sequential design of experiments with heterogeneous recurrent event endpoints. Xia and Hoover (2007) described a group sequential framework for Poisson outcome variables when interim analyses were timed after landmark numbers of events had occurred in each arm. They based their testing framework on the exact distribution of the number of events from one arm, conditional on the total observed number of events. Cook et al. (2010) presented methods for the sequential analysis of data from experiments with recurrent event responses observed over two periods, where one is a baseline period of observation. Recently, Mutze et al. (2018a) also considered recurrent event responses, presenting group sequential procedures for a robust semi-parametric analysis of such data. Finally, Mutze et al. (2018b) described methodology for the group sequential design of trials with negative-binomial outcomes based on Wald test statistics using maximum likelihood estimators.

Here, we focus on a different design scenario to these articles, in which each outcome is assumed to be a single Poisson distributed variable, with its precise distribution dependent only on which arm it was accrued from. First, we describe how established group sequential design theory can be applied in this setting, allowing widely available software for design determination to be employed, and operating characteristics to be approximately controlled. Following this, we detail a novel design that allows for the exact computation of a design’s operating characteristics based on the extension of the approach of Menon et al. (2011) to group sequential experiments. To allow maximal efficiency gains to be attained from the group sequential tests, we then describe an effective means of choosing stopping boundaries in a near-optimal manner. Next, we expound on the potential gains a group sequential design could bring through two hypothetical examples, before concluding with a discussion of the advantages and disadvantages of the two described procedures.

## 2 Methods

### 2.1 Notation and hypotheses

We consider an experiment in which data is to be accrued from two arms, which we index by *j* ∈ {1, 2}. In addition, we suppose that our group sequential experiment will have at most *K* ∈ ℕ^+^ stages (explicitly permitting *K* = 1, which corresponds to a fixed sample design), and we index the stages by *k* ∈ {1, …, *K*}. Thus, from here, unless stated otherwise, it can be assumed that *j* ∈ {1, 2} and *k* ∈ {1, …, *K*}.

We then denote our outcomes variables by *X_ijk_*: the number of observed events for sample *i*, in arm *j*, in stage *k*. And, we assume that each outcome variable is accrued following observation over an equal fixed positive time, and that then *X_ijk_* ~ *Po*(*λ_j_*), for *λ_j_* ∈ ℝ^+^ the mean event rate on arm *j*. Furthermore, for simplicity, we assume that analyses are performed after *kn*, *n* ∈ ℕ^+^, outcome variables have been gathered on each of the two arms (implying *i* ∈ {1, …, *n*}). Note however that the methods which follow could readily be extended to consider unequal allocation to the arms in and across the stages.

Our null hypothesis of interest will be *H*_0_ : *λ*_1_ = *λ*_2_ ∈ Λ_0_ ⊂ (0, ∞). That is, we test a composite null hypothesis, to expressly allow for design in scenarios (see Example 1 below) in which a range of possible values of the null mean response are anticipated. Moreover, we power our experiment for a scenario in which *λ*_1_ = *λ*_2_ + *δ* ∈ Λ_1_ ⊂ (0, ∞) for *δ* ∈ ℝ^+^, as we are principally motivated by a clinical scenario in which we hope the novel treatment (arm *k* = 2) will reduce the mean response. However, note that a design for *δ* ∈ ℝ^−^ could be identified similarly.

We allow early stopping both to reject and to not reject *H*_0_, with our stopping rules dependent on vectors of boundaries that we denote by ***a*** = (*a*_1_, …, *a_K_*) and ***r*** = (*r*_1_, …, *r_K_*). For now, we assume that ***a***, ***r*** ∈ ℝ^*K*^. More precisely, we will specify test statistics *T_k_*, which will be used with the following stopping rules

- For *k* ∈ {1, … , *K*−1}
  - If *T_k_* ≥ *r_k_* stop the experiment, rejecting *H*_0_;
  - If *T_k_* < *a_k_* stop the experiment, not rejecting *H*_0_;
  - If *T_k_* ∈ [*a_k_*, *r_k_*) continue the experiment to stage *k* + 1.
- For *k* = *K*
  - If *T_k_* ≥ *r_k_* stop the experiment, rejecting *H*_0_;
  - If *T_k_* < *a_k_* stop the experiment, not rejecting *H*_0_.

To ensure that our stopping rules make sense (i.e., that we do not simultaneously recommend to both reject and not reject *H*_0_), and to guarantee that a conclusion is drawn on *H*_0_, we enforce that *a_k_* < *r_k_* for *k* ∈ {1, …, *K*−1} and set *a_K_* = *r_K_*. We will also make use of the notation ***a**_k_* = (*a*_1_, …, *a_k_*), and similarly for ***r***.

Key to the identification of our group sequential designs will then be the ability to calculate the probability we stop to reject *H*_0_, *R_k_*(·), or stop not rejecting *H*_0_, *A_k_*(·), at each analysis *k*, conditional on the values of the design parameters. Formally, these are

$$A_{k}\left(\lambda_{1},\lambda_{2}|n,{\text{a}}_{k},{\text{r}}_{k}\right)=\{\begin{array}{ll} \mathbb{P}\left[T_{1}\in \left(-\infty ,a_{1}\right)|\lambda_{1},\lambda_{2},n\right] & :k=1,\\ \mathbb{P}\{T_{1}\in [a_{1},r_{1}),\ldots ,T_{k-1}\in [a_{k-1},r_{k-1}), & \\ T_{k}\in \left(-\infty ,a_{k}\right)|\lambda_{1},\lambda_{2},n\} & :k\in \left\{2,\ldots ,K\right\} \end{array}$$

and

$$R_{k}\left(\lambda_{1},\lambda_{2}|n,{\text{a}}_{k},{\text{r}}_{k}\right)=\{\begin{array}{ll} \mathbb{P}\left\{T_{1}\in \left[r_{1},\infty \right)|\lambda_{1},\lambda_{2},n\right\} & :k=1,\\ \mathbb{P}\{T_{1}\in [a_{1},r_{1}),\ldots ,T_{k-1}\in [a_{k-1},r_{k-1}), & \\ T_{k}\in \left[r_{k},\infty \right)|\lambda_{1},\lambda_{2},n\} & :k\in \left\{2,\ldots ,K\right\} \end{array}$$

respectively, where we note that the distribution of the *T_k_* will be dependent upon *λ*_1_, *λ*_2_, and *n*.

With the above, we can compute the maximal type-I error-rate of our test as

$$\alpha^{\prime }\left(n,\text{a},\text{r}\right)=\underset{\lambda \in \Lambda_{0}}{\text{max}}\sum_{k=1}^{K}R_{k}\left(\lambda ,\lambda |n,{\text{a}}_{k},{\text{r}}_{k}\right)$$

Furthermore, the maximal type-II error-rate is

$$\beta^{\prime }\left(n,\text{a},\text{r}\right)=\underset{\lambda \in \Lambda_{1}}{\text{max}}\sum_{k=1}^{K}A_{k}\left(\lambda ,\lambda -\delta |n,{\text{a}}_{k},{\text{r}}_{k}\right)$$

Consequently, our goal in what follows will be to choose values for *n*, ***a***, and ***r*** such that *α*′(*n*, ***a***, ***r***) ≤ *α*, and *β*′(*n*, ***a***, ***r***) ≤ *β*, for specified *α*, *β* ∈ (0, 1).

### 2.2 Design based on a normal approximation

First, we describe how we can compute group sequential designs based on the asymptotic distribution of Wald-type test statistics. To this end, denote the maximum likelihood estimate of *λ_j_*, at analysis *k*, by ${\hat{\lambda }}_{jk}.$ We have

$${\hat{\lambda }}_{jk}=\frac{1}{kn}\sum_{l=1}^{k}\sum_{i=1}^{n}X_{ijl}$$

Next, note that the expected Fisher information of the parameter *λ_j_* at analysis *k* is *I_jk_* = *kn*/*λ_j_*. Then, key to design determination is the notion of the information level at analysis *k*, which serves a measure of the knowledge available about the difference of the means of the two treatment arms. We denote this information by *ℐ*_*k*_ with it given by

$${ℐ}_{k}=\frac{1}{\frac{1}{I_{1k}}+\frac{1}{I_{2k}}}=\frac{kn}{\lambda_{1}+\lambda_{2}}$$

Importantly, we have

$$\left({\hat{\lambda }}_{1k}-{\hat{\lambda }}_{2k}\right)\sqrt{{ℐ}_{k}}\overset{𝒟}{\to }N\left(0,1\right)\;\text{as}\;n\to \infty$$

Unfortunately, the information level *ℐ_k_* depends on the unknown means, *λ*_1_ and *λ*_2_. Therefore, to test *H*_0_ using asymptotic normality we replace *ℐ_k_* by a consistent estimator, ${\hat{ℐ}}_{k},$ obtaining the Wald-type test statistic for analysis *k* as

$$T_{Wk}=\left({\hat{\lambda }}_{1k}-{\hat{\lambda }}_{2k}\right)\sqrt{{\hat{ℐ}}_{k}}=\left({\hat{\lambda }}_{1k}-{\hat{\lambda }}_{2k}\right)\sqrt{\frac{kn}{{\hat{\lambda }}_{1k}+{\hat{\lambda }}_{2k}}}$$

Now, because *ℐ_k_* is estimated using a consistent estimator, Slutsky’s theorem tells us that *T_Wk_* is still asymptotically normally distributed. Moreover, by standard results in group sequential design theory, the vector of Wald-type test statistics ***T**_W_* = (*T*_*W*1_, …, *T_WK_*) follows what has been referred to as the ‘canonical joint distribution’ (Scharfstein et al. 1997; Jennison and Turnbull 2000). That is, ***T**_W_* is asymptotically multivariate normal with mean vector $\left(\lambda_{1}-\lambda_{2}\right){ℐ}_{K}^{1/2}=\left(\lambda_{1}-\lambda_{2}\right){\left({ℐ}_{1}^{1/2},\ldots ,{ℐ}_{K}^{1/2}\right)}^{⊤}$ and *K* × *K* covariance matrix Σ = {Σ_*k*_1_*k*_2__} with $\Sigma_{k_{1}k_{2}}=\Sigma_{k_{2}k_{1}}=\sqrt{{ℐ}_{k_{1}}/{ℐ}_{k_{2}}}=\sqrt{k_{1}/k_{2}},$ for *k*_2_ ∈ {1, …, *K*} and *k*_1_ ∈ {1, …, *k*_2_}.

Accordingly, we can compute the *A_k_*(·) and *R_k_*(·) via

$$A_{k}\left(\lambda_{1},\lambda_{2}|n,{\text{a}}_{k},{\text{r}}_{k}\right)=\{\begin{array}{ll} \int_{-\infty }^{a_{1}}\phi \left[x,\left(\lambda_{1}-\lambda_{2}\right){ℐ}_{1}^{1/2},1\right]\text{d}x & :k=1,\\ \int_{a_{1}}^{r_{1}}\ldots \int_{-\infty }^{a_{k}}\phi \left[\text{x},\left(\lambda_{1}-\lambda_{2}\right){ℐ}_{k}^{1/2},\Sigma_{k}\right]\text{d}x_{k}\ldots \text{d}x_{1} & :k\in \left\{2,\ldots ,K\right\} \end{array}$$

and

$$R_{k}\left(\lambda_{1},\lambda_{2}|n,{\text{a}}_{k},{\text{r}}_{k}\right)=\{\begin{array}{ll} \int_{r_{1}}^{\infty }\phi \left[x,\left(\lambda_{1}-\lambda_{2}\right){ℐ}_{1}^{1/2},1\right]\text{d}x & :k=1,\\ \int_{a_{1}}^{r_{1}}\ldots \int_{r_{k}}^{\infty }\phi \left[\text{x},\left(\lambda_{1}-\lambda_{2}\right){ℐ}_{k}^{1/2},\Sigma_{k}\right]\text{d}x_{k}\ldots \text{d}x_{1} & :k\in \left\{2,\ldots ,K\right\} \end{array}$$

respectively. Here, *ϕ*(***x***, ***μ***, **Ω**) is the probability density function of a multivariate normal distribution with mean vector ***μ*** and covariance matrix **Ω**, and Σ*_k_* signifies the restriction of Σ to its first *k* rows and columns.

### 2.3 Design based on exact calculations

Given that the methodology described in Section 2.2 relies upon asymptotic theory, the operating characteristics computed using multivariate normal distribution functions could be a poor approximation to their empirical values. Therefore, to permit strict error control we now extend the results of Menon et al. (2011) to group sequential designs. To achieve this, note that the sum of the outcomes in arm *j* in stage *k*, *Y_jk_* say, has the following distribution based on the familiar result that the sum of independent Poisson random variables is itself Poisson

$$Y_{jk}=\sum_{i=1}^{n}X_{ijk}∼Po\left(n\lambda_{j}\right)$$

Then, the difference in the sum of the outcomes on each arm, ${\tilde{T}}_{k}=Y_{1k}-Y_{2k},$ as a difference between two independent Poisson random variables, has a Skellam distribution (Skellam 1946). We signify this by ${\tilde{T}}_{k}∼Skellam\left(n\lambda_{1},n\lambda_{2}\right),$ and denote the probability mass and cumulative distribution functions of ${\tilde{T}}_{k}$ on its support ℤ as follows

g(t|n,λ1,λ2)=ℙ(T˜k=t|n,λ1,λ2)=e−n(λ1+λ2)(λ1λ2)t2I|t|(2nλ1λ2),G(t|n,λ1,λ2)=ℙ(T˜k≤t|n,λ1,λ2)=∑{s∈ℤ:s≤t}g(s|n,λ1,λ2)

where *I_ν_*(·) is the modified Bessel function of the first kind, and we make use of the particular representation presented in Menon et al. (2011).

Our test statistic at analysis *k* for the exact design is then $T_{Sk}={\tilde{T}}_{1}+\cdots +{\tilde{T}}_{k}.$ Now, within the context of exact group sequential designs for Bernoulli distributed outcome variables, it is well understood that the interim test statistics do not in general have a simple distribution (Jung 2012). Similarly, it is important to note that *T_Sk_* ≁ *Skellam*(*knλ*_1_, *knλ*_2_). What is more, unlike the case for Bernoulli outcomes, we are in fact unable to compute the probability mass function of *T_Sk_* across its entire support, ℤ. This is a consequence specifically of the fact that the support of each ${\tilde{T}}_{k}$ is infinite. However, we can compute a part of the probability mass function, which we denote by *h_k_*(*t*|*n*, *λ*_1_, *λ*_2_, ***a**_k_*, ***r**_k_*) = ℙ(*T_Sk_* = *t*|*n*, *λ*_1_, *λ*_2_, ***a**_k_*, ***r**_k_*). Explicitly

$$h_{k}\left(t|n,\lambda_{1},\lambda_{2,}{\text{a}}_{k,}{\text{r}}_{k}\right)=\{\begin{array}{ll} g\left(t|n,\lambda_{1},\lambda_{2}\right) & :k=1,\\ \sum_{s=\left\lfloor a_{k-1}\right\rfloor }^{\left\lfloor r_{k-1}\right\rfloor -1}h_{k-1}\left(s|n,\lambda_{1},\lambda_{2},{\text{a}}_{k-1,}{\text{r}}_{k-1}\right) & \\ \;g\left(t-s|n,\lambda_{1},\lambda_{2}\right) & :k\in \left\{2,\ldots ,K-1\right\},t\in \left[a_{k},r_{k}\right) \end{array}$$

That is, we are able to evaluate the probability mass function for *T_Sk_* ∈ [*a_k_*, *r_k_*). This result is in essence an extension of that of Schultz et al. (1973) for single-arm experiments with Bernoulli outcome variables.

Fortunately, the above is all that is required to compute *A_k_*(·) and *R_k_*(·), because

$$R_{k}\left(\lambda_{1},\lambda_{2}|n,{\text{a}}_{k,}{\text{r}}_{k}\right)=\{\begin{array}{ll} 1-G\left(r_{1}-1|n,\lambda_{1},\lambda_{2}\right) & :k=1,\\ \sum_{t=\left\lfloor a_{k-1}\right\rfloor }^{\left\lfloor r_{k-1}\right\rfloor -1}h_{k-1}\left(t|n,\lambda_{1},\lambda_{2},{\text{a}}_{k-1,}{\text{r}}_{k-1}\right) & \\ \;\{1-G\left(r_{k}-t-1|n,\lambda_{1},\lambda_{2}\right)\} & :k\in \left\{2,\ldots ,K\right\} \end{array}$$

and

$$A_{k}\left(\lambda_{1},\lambda_{2}|n,\text{a},\text{r}\right)=\{\begin{array}{ll} G\left(a_{1}-1|n,\lambda_{1},\lambda_{2}\right) & :k=1,\\ \sum_{t=\left\lfloor a_{k-1}\right\rfloor }^{\left\lfloor r_{k-1}\right\rfloor -1}h_{k-1}\left(t|n,\lambda_{1},\lambda_{2},{\text{a}}_{k-1,}{\text{r}}_{k-1}\right) & \\ \;G\left(a_{k}-t-1|n,\lambda_{1},\lambda_{2}\right) & :k\in \left\{2,\ldots ,K\right\} \end{array}$$

Thus, we can compute the operating characteristics to arbitrary accuracy provided we are able to evaluate *g*(·) and *G*(·). We can achieve this in R using the skellam package (Lewis et al. 2016), with *G*(·) in particular computed using a relationship between the Skellam and *χ* ^2^ distributions due to Johnson (1959).

### 2.4 Boundary determination

A variety of methods have been presented in the literature through which stopping boundaries and sample sizes for group sequential designs can be determined. For the method of Section 2.2, the normal approximation approach, many of these could be applied in our considered Poisson-outcome experimental design scenario. This includes comparatively simpler procedures such as that of Pampallona and Tsiatis (1994), through to more complex methods that allow design optimization (Wason et al. 2012). For our framework from Section 2.3, however, there are fewer methods available that could be directly applied to determine suitable designs. The primary reason for this is that in this setting the stopping boundaries are best treated as discrete parameters (i.e., it is best to apply the restriction ***a***, ***r*** ∈ ℤ^*K*^), otherwise a redundancy will exist in the design space (e.g., two designs that are otherwise equal apart from their values of *a*_1_ being 2.1 and 2.2 respectively would have the same operating characteristics).

This makes a brute-force approach that searches across possible designs tempting, similar to that which has been applied to both non-randomized and randomized trials with binary outcome variables. Unfortunately, such a solution will for many *K* be computationally expensive given that the design space is 2 *K*-dimensional. Consequently, here, we propose an approach to determining ‘near-optimal’ values for these discrete bounds based on the error spending approach to group sequential design (Lan and DeMets 1983). We also utilize this method for determining designs based on the normal approximation procedure, in order to allow for a fairer comparison to the performance of the exact procedure, and as in our experience it offers an advantageous tradeoff between computational run-time and design efficiency.

However, for the exact approach with *K* = 2, we do also contrast the performance of these near-optimal designs to ‘optimal’ designs identified via an extensive search over possible values for *n*, ***a*** and ***r***. Through this, we aim to demonstrate that a brute-force style approach may often provide little advantage over the comparatively easy-to-identify near-optimal designs. For this search, note that the infinite support of the ${\tilde{T}}_{Sk}$ implies that an exhaustive assessment of all possible boundary values is impossible. Accordingly, for any *n*, a method is required for limiting the considered ***a*** and ***r*** in a logical manner. Note that these restrictions should take in to account the chosen values for Λ_0_, Λ_1_, and *δ*. Here, for any *n* our approach is to identify the solutions of the following equations

$$\begin{array}{l} a_{*}=\underset{a\in \mathbb{Z}}{\text{argmax}}\left[\underset{\lambda \in \Lambda_{0}}{\text{max}}\left\{A_{1}\left(\lambda ,\lambda |n,a,r\right)\leq \epsilon \right\}\right],\\ r_{*}=\underset{r\in \mathbb{Z}}{\text{argmin}}\left[\underset{\lambda \in \Lambda_{1}}{\text{max}}\left\{R_{1}\left(\lambda ,\lambda -\text{δ}|n,a,r\right)\leq \epsilon \right\}\right] \end{array}$$

That is, we choose *r*_∗_ as the minimal integer that ensures the probability of stopping the trial after stage one to reject *H*_0_, under *λ*_1_ = *λ*_2_ + *δ* ∈ Λ_1_, is at most *ϵ*, and similarly for *a*_∗_. We then search over boundaries such that *a*_1_ ∈ [*a*_∗_, *r*_∗_−2], *r*_1_ ∈ [*a*_1_ + 2, *r*_∗_], and *a*_2_ ∈ [*a*_1_ + *a*_∗_, *r*_1_ + *r*_∗_]. This ensures that having continued to stage two, the conditional probability of rejecting *H*_0_ is at most *ϵ* when *λ*_1_ = *λ*_2_ + *δ* ∈ Λ_1_, with a similar statement holding true for the probability of not rejecting *H*_0_ at the end of stage two when *λ*_1_ = *λ*_2_ ∈ Λ_0_. Thus, we allow values for ***r*** that provide conditional probabilities of stopping after each stage to reject *H*_0_ of at least *ϵ* under some scenario for which we wish to power the trial, and similarly for the values of ***a***. For, if *ϵ* ≪ 1 it is reasonable to expect that little could be gained from designs that are not captured as part of this search procedure. Accordingly, in our search we set *ϵ* = 10^−7^. Then, we perform our evaluations over group-sizes *n* such that *n* ∈ {1, …, 1.5*n*_fixed_}, where *n*_fixed_ is the group-size required by a corresponding fixed-sample (*K* = 1) design. Note that the maximal type-I and type-II error-rates for any considered design, across the sets Λ_0_ and Λ_1_, are identified using Brent’s algorithm (Brent 1973).

In contrast, using the error-spending approach, we specify error spending vectors ***π**_A_* = (*π*_*A*1_, …, *π_AK_*) and ***π**_R_* = (*π*_*R*1_, …, *π_RK_*), with

$$\sum_{k=1}^{K}\pi_{Rk}=\text{α},\;\sum_{k=1}^{K}\pi_{Ak}=\beta ,\;\pi_{Rk,}\pi_{Ak}\geq 0,k\in \left\{1,\ldots ,K\right\}$$

that then imply particular stopping boundaries, and a particular required group size. First, we describe how the boundaries are chosen for a fixed *n* ∈ ℕ^+^, as well as fixed ***π**_A_* and ***π**_R_* conforming to the requirements above. We then describe how *n* can subsequently be chosen for these ***π**_A_* and ***π**_R_*, before describing how the spending vectors themselves can be specified.

Put simply, for a given *n*, ***π**_A_* and ***π**_R_*, we recursively identify the boundaries, beginning with *r*_1_. The precise nature of the calculations differ for the exact and normal approximation based approaches. We proceed by describing the differences before expanding on the reasons that they are present.

First, for the exact procedure, for *k* ∈ {1, …, *K*−1}, we iterate between finding *r_k_* and *a_k_* as the solutions to

$$\underset{r_{k}\in \mathbb{Z}}{\text{argmin}}\left[\underset{\lambda \in \Lambda_{0}}{\text{max}}\;R_{k}\left(\lambda ,\lambda |n,{\text{a}}_{k-1},{\text{r}}_{k}\right)\leq \pi_{Rk}\right]$$

and

$$\underset{a_{k}\in \mathbb{Z}}{\text{argmax}}\left[\underset{\lambda \in \Lambda_{1}}{\text{max}}\;A_{k}\left(\lambda ,\lambda -\delta |n,{\text{a}}_{k},{\text{r}}_{k-1}\right)\leq \pi_{Ak}\right]$$

respectively, where we may arbitrary take ***a***_0_ = ***r***_0_ = 0 since these are not used in our evaluations of *A*_1_(·) and *R*_1_(·).

Whilst, for the normal approximation approach, for *k* ∈ {1, …, *K*−1}, we iterate between finding *r_k_* and *a_k_* as the solutions to

$$\underset{r_{k}\in \mathbb{R}}{\text{argmin}}\left\{R_{k}\left(\lambda ,\lambda |n,{\text{a}}_{k-1},{\text{r}}_{k}\right)\leq \pi_{Rk}\right\}$$

for some arbitrarily chosen *λ* ∈ Λ_0_, and

$$\underset{a_{k}\in \mathbb{R}}{\text{argmax}}\left[A_{k}\left\{\text{sup}\left(\Lambda_{1}\right),\text{sup}\left(\Lambda_{1}\right)-\delta |n,{\text{a}}_{k},{\text{r}}_{k-1}\right\}\leq \pi_{Ak}\right]$$

In either case, we then utilize the formula to specify *r_K_*, but not *a_K_*, setting it instead as *a_K_* = *r_K_*. This ensures that we control the theoretical type-I error-rate to the desired level *α*, and as discussed earlier it guarantees that a decision is made on *H*_0_ during the course of the experiment.

Now, we note the reasons for the differences in the approaches utilized for the two methods. They arise because of our desire to control the type-I and type-II error-rates over the sets Λ_0_ and Λ_1_ respectively. Specifically, for the normal approximation approach, note that the distribution of the ***T**_W_* when *λ*_1_ = *λ*_2_ ∈ Λ_0_ does not depend on the specific shared value of *λ*_1_ and *λ*_2_. Thus, the type-I error-rate can be found for an arbitrarily chosen value of these parameters, and in turn we need only base our values *r_k_* on controlling the *R_k_*(·) to below *π_Rk_* for this arbitrary value. Similarly, the theoretical type-II error-rate for the normal approximation approach when *λ*_1_ = *λ*_2_ + *δ* ∈ Λ_1_ is maximized when *λ*_1_ = sup(Λ_1_), since this provides the minimal possible information over the set Λ_1_. Thus, if we wish to control our type-II error-rate to at most *β* over all possible scenarios *λ*_1_ = *λ*_2_ + *δ* ∈ Λ_1_, we can simply choose our *a_k_* to constrain the *A_k_*(·) to at most *π_Ak_* when *λ*_1_ = *λ*_2_ + *δ* = sup(Λ_1_).

In contrast, for the exact test the maximal type-I and type-II error-rates are not easy to identify. For, we may hope that an analytical formulae for the location of the maximal error-rates could be derived (e.g., by proving monotonicity of rejection probabilities across the sets Λ_0_ and Λ_1_). However, as we discuss further in the Supplementary Material, we believe it is unlikely that this could be achieved, given that such a result was only recently derived for the comparatively simple case of a single-arm trial with Bernoulli outcomes (Shan et al. 2017), and at least for the type-I error-rate our explorations suggest there is no simple pattern to the location of the maxima. For this reason our determination of the *a_k_* and *r_k_* retains a one-dimensional numerical search for the maximal values of *A_k_*(·) and *R_k_*(·) respectively. With this, however, we are guaranteed to control the error-rates to the desired level, whilst the empirical error-rates of normal approximation designs may be above their nominal levels.

The above completes our computation of the stopping boundaries for fixed *n*, ***π**_A_* and ***π**_R_*. For any such error spending vectors, the value of *n* that provides the desired power can then be determined as

$$\begin{array}{c} \underset{n\in {\mathbb{N}}^{+}}{\text{argmin}}\left\{\beta^{\prime }\left(n,\text{a},\text{r}\right)\leq \beta \right\} \end{array}$$

where the ***a*** and ***r*** here are those specifically derived for the particular *n* under assessment to control the type-I error-rate, using the methods above.

Thus, by the above we are able to determine the *n*, ***a***, and ***r*** that correspond to any choice of ***π**_A_* and ***π**_R_*. Near-optimal designs are then identified by searching over possible choices for these two error spending vectors: by searching over a large number of such vectors, an approximately exhaustive search over possible designs can be executed.

All that is additionally required to choose a design is an optimality criteria to choose amongst potential designs. Here, we use the following, which has been considered extensively in the past for similar group sequential design settings (Mander et al. 2012; Wason et al. 2012)

$$w_{1}ESS\left(\lambda_{\text{ESS}},\lambda_{\text{ESS}}|n,\text{a},\text{r}\right)+w_{2}ESS\left(\lambda_{\text{ESS}},\lambda_{\text{ESS}}-\delta |n,\text{a},\text{r}\right)+w_{3}2Kn$$

where *ESS*(·) is a function that computes the ESS, given by

$$ESS\left(\lambda_{1},\lambda_{2}|n,\text{a},\text{r}\right)=2n\sum_{k=1}^{K}k\left[A_{k}\left(\lambda_{1,}\lambda_{2}|\;n,{\text{a}}_{k},{\text{r}}_{k}\right)+R_{k}\left(\lambda_{1},\lambda_{2}|\;n,{\text{a}}_{k},{\text{r}}_{k}\right)\right]$$

and *λ*_ESS_ is a specified mean response in arm 1. Furthermore, the *w_l_* ∈ [0, ∞), *l* ∈ {1, 2, 3}, are then weights given to the different components of the optimality function. Note we should generally ensure that *w*_1_ + *w*_2_ ∈ (0, ∞) as multiple designs will often have the same minimal maximal sample size, 2*Kn*. For brevity in what follows, we set ***w*** = (*w*_1_, *w*_2_, *w*_3_).

## 3 Results

### 3.1 Example group-sequential designs

To demonstrate the potential efficiency gains from utilizing a group-sequential design, and to compare the exact and normal approximation approaches, we consider two example trial design scenarios. The results for Example 1 are presented below, whilst those for Example 2 are included in the Supplementary Material.

We motivate the design parameters for Example 1 by considering a hypothetical obstructive sleep apnea-hypopnea (OSAH) trial. Specifically, continuous positive airway pressure is typically the first line treatment for severe sufferers of OSAH (National Institute for Health and Care Excellence 2008). However, its benefits in milder disease are less certain (Patel et al. 2003; Weaver et al. 2012), and intolerance of continuous positive airway pressure is also common (Weaver and Grunstein 2008). Therefore, we consider the design of a trial that aims to examine the efficacy of an alternative treatment option for OSAH in more moderate disease cases, such as for example the oral mandibular advancement devices that were examined by Quinnell et al. (2014) amongst others. Thus arm 1 will correspond to no treatment, and arm 2 to the new treatment option of interest.

We assume that, as is common in OSAH studies, the apnea-hypopnea index (AHI, the combined average number of apneas and hypopneas that occur per hour of sleep) will be the primary endpoint of interest. To account for variability in the mean AHI of enrolled patients on the control arm, we specify Λ_0_ = Λ_1_ = [15, 30] (corresponding to the established definition of moderate OSAH disease). Finally, we assume that it is desired to control the type-I error-rate to *α* = 0.05 and have power of 1−*β* = 0.8 when *δ* = 2.25 (corresponding to at least a 15% reduction in AHI for moderate disease sufferers).

We consider the optimal designs for

$$\begin{array}{l} {\text{w}}_{1}=\left(1,0,0\right),{\text{w}}_{2}=\left(0,1,0\right),{\text{w}}_{3}=\left(1/2,1/2,0\right),\\ {\text{w}}_{4}=\left(1/2,0,1/2\right),{\text{w}}_{5}=\left(0,1/2,1/2\right),{\text{w}}_{6}=\left(1/3,0,1/3\right) \end{array}$$

when *K* ∈ {2, 3}, taking *λ*_ESS_ = 15 as an example.

Finally, for the case *K* = 2, when utilizing the error-spending approach, we consider all combinations of ***π**_A_* and ***π**_R_*, conforming to our requirements from earlier, with (*π*_*A*1_, *π*_*R*1_) ∈ {0.02, 0.04, …, 0.16, 0.18} × {0.005, 0.01, …, 0.045}. Similarly, for *K* = 3 we examine all permissible combinations with (*π*_*A*1_, *π*_*A*2_, *π*_*R*1_, *π*_*R*2_) ∈ {0.03, 0.06, 0.09, 0.12}^2^ × {0.01, 0.015, …, 0.035}^2^. Note that code to reproduce our results is available from https://github.com/mjg211/article_code.

Now, the optimal designs for *K* ∈ {2, 3}, amongst the considered ***π**_A_* and ***π**_R_*, and using the (approximately) exhaustive search to determine optimal designs, were determined for the six stated values of ***w***. They are displayed, along with the corresponding single-stage designs based on the exact and normal approximation methods, in Table 1.

We observe that, as would be expected, utilizing a group sequential approach reduces the ESS when *λ*_1_ = *λ*_2_ = *λ*_ESS_ and when *λ*_1_ = *λ*_2_ + *δ* = *λ*_ESS_ relative to using a single-stage design. In particular, the ESS when *λ*_1_ = *λ*_2_ = *λ*_ESS_ can be reduced by as much as 41% and 44% when using the normal approximation or exact approaches respectively (for *K* = 3, compared to their respective required sample sizes when *K* = 1).

Moreover, as is typical for group sequential designs, increasing the value of *K* allows us to increase efficiency further in terms of the ESS, but this comes at a cost to the maximal possible sample sizes. Interestingly though, using either design approach, there do exist designs that require only very minor increases to the maximal possible sample size that bring sizeable reductions to the considered ESSs. In particular, the optimal designs for ***w***_6_, which place a non-zero weight on each of the three components of the optimality criteria, appear to perform particularly well in comparison to a single-stage approach under both *λ*_1_ = *λ*_2_ = *λ*_ESS_ and *λ*_1_ = *λ*_2_ + *δ* = *λ*_ESS_, without requiring a substantial increase to the maximal possible sample size.

Finally, observe that the optimal design search is able to identify for each considered value of ***w*** a design that out-performs the corresponding near-optimal design. However, the difference between the operating characteristics of these designs is typically small. For example, for ***w***_1_, the optimal design has *ESS*(*λ*_ESS_, *λ*_ESS_) = 92.8, whilst the near-optimal design has *ESS*(*λ*_ESS_, *λ*_ESS_) = 94.6, an increase of only 2%.

### 3.2 Empirical performance of normal approximation designs

Here, we expand on the potential problems associated with use of the normal approximation approach, utilizing simulation to assess the difference between the theoretical (estimated) error-rates of normal approximation designs in comparison to their empirical values. Specifically, to examine in detail the potential differences between the estimated and empirical values, we identified the normal-approximation error-spending designs for Λ_0_ = Λ_1_ = *λ*_0_ ∈ {1, 1.5, …, 9.5, 10}, when *δ* ∈ {0.25*λ*, 0.275*λ*, …, 0.725*λ*, 0.75*λ*}, ***π**_A_* = (0.1, 0.1), and ***π**_R_* = (0.025, 0.025). For each of these 399 designs, 100,000 simulations were then used to evaluate the empirical type-I and type-II error-rates for comparison to their values estimated via the formulae of Section 2.2.

**Figure 1:**
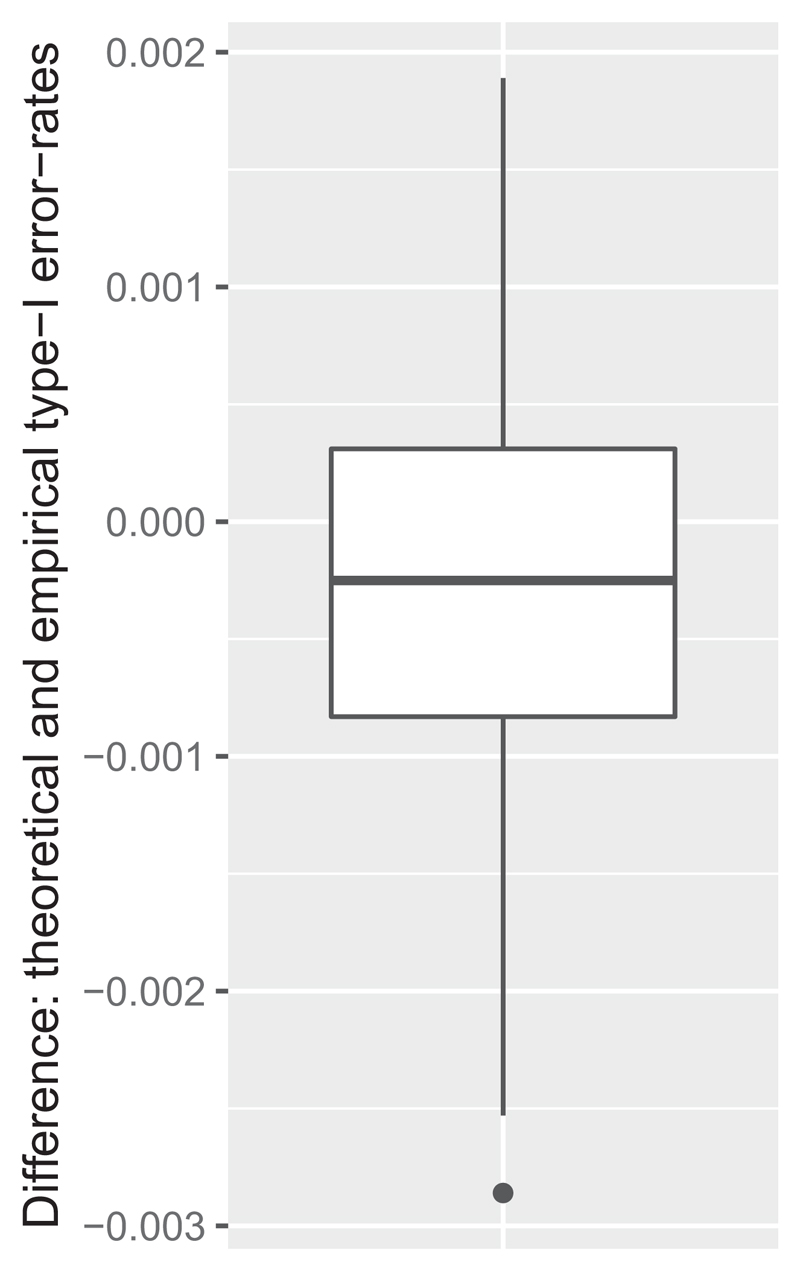
The difference between the theoretical and empirical type-I (*λ*_1_ = *λ*_2_) error-rate of exact designs is shown.

**Figure 2:**
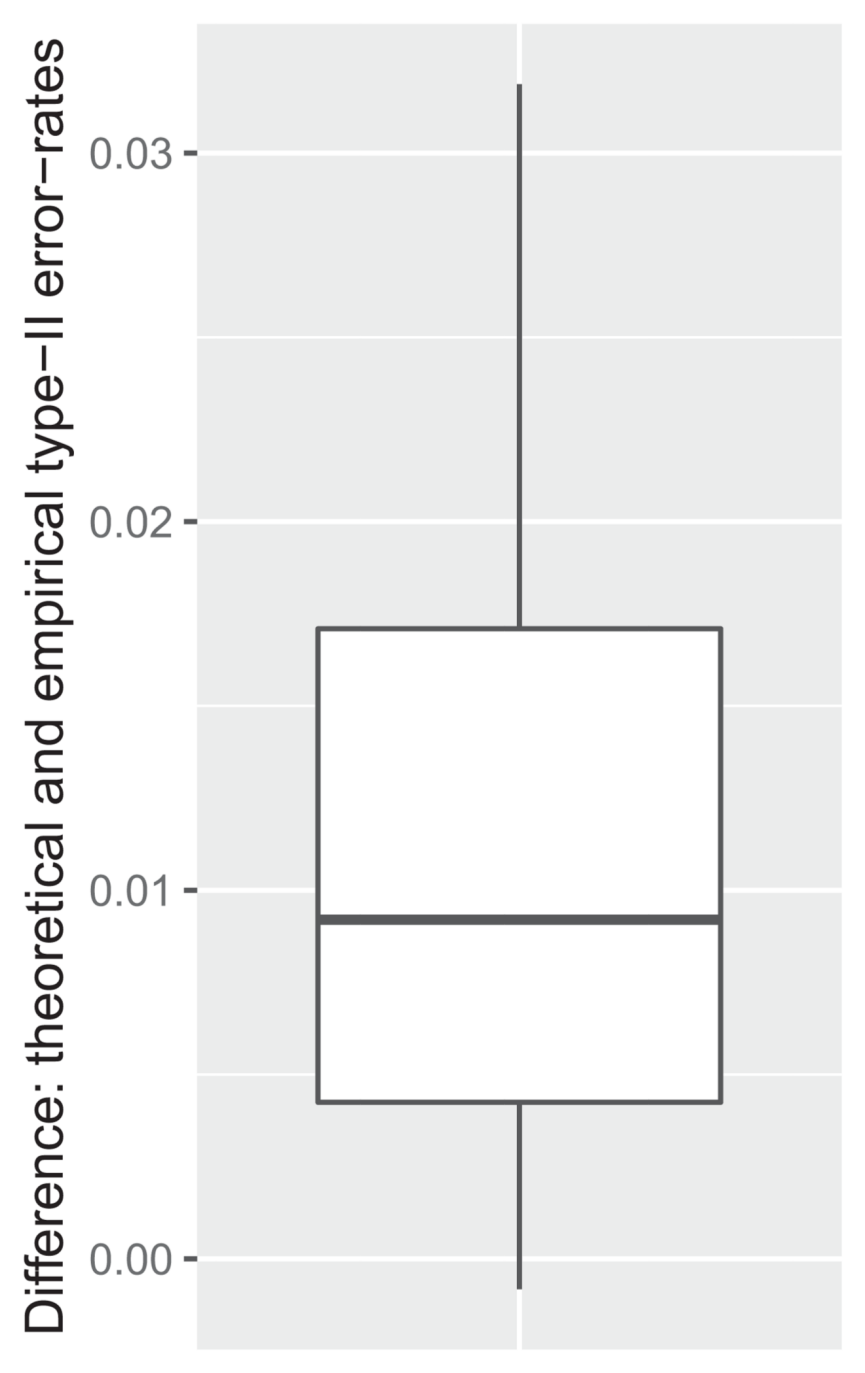
The difference between the theoretical and empirical type-II (*λ*_1_ = *λ*_2_ + *δ*) error-rate of exact designs is shown.

The difference between the estimated and empirical type-I and type-II error-rates of the designs are presented in Figures 1 and 2. In general, the differences are small on the depicted raw scale, with the maximal absolute difference in type-I and type-II error-rates being 0.0029 and 0.0319 to 4 dp respectively. However, when considered as a percentage difference from the estimated value, the maximal differences are 5.8% and 24.4%. These figures, along with other considerations, impact the applicability of the normal approximation approach.

## 4 Discussion

Here, we have described how we can use well established methodology to design group sequential experiments with Poisson distributed outcomes. Furthermore, in order to provide a method that is not dependent on asymptotic theory, we also utilized the Skellam distribution to determine operating characteristics exactly. This exact design required, in particular, careful consideration of how to evaluate the probability of stopping at each interim analysis.

Furthermore, to permit efficient group sequential designs to be identified for any value of *K*, we presented a method for design determination based on the error spending approach to group sequential design. Specifically, we searched over possible error spending vectors to find which amongst these minimized a particular optimality function. Whilst this is unlikely to identify the best possible design, we believe it is an effective means of finding efficient designs, particularly if parallelization is used to search over a large number of possible spending vectors. Indeed, from Table 1 we observed that at least for Example 1, the near-optimal designs for *K* = 2 where only slightly less efficient than the optimal designs identified for an extensive search over possible values of *n*, ***a***, and ***r***.

Overall, for our considered example, we demonstrated that using a group sequential design had the potential to improve efficiency substantially; with the ESS for *λ*_1_ = *λ*_2_ = *λ*_ESS_ in Example 1 reducible by as much as 44%. This unfortunately came at a cost of an increase to the maximal possible sample size of 23%. However, we did identify several designs that required only minimal increases to the maximal possible sample size, whilst still reducing the ESS when *λ*_1_ = *λ*_2_ = *λ*_ESS_ and when *λ*_1_ = *λ*_2_ + *δ* = *λ*_ESS_ notably. Statistically speaking, such designs have almost no disadvantages. Furthermore, as is typical with group sequential designs, the identified efficiency gains increased with the value of *K*. Note that we presented results here for *K* ∈ {2, 3}, as our investigations have suggested the value of setting *K* > 3, in terms of reducing the ESS, is often small. This should not be interpreted as design determination for *K* > 3 being intractable.

Note that one limitation of our sequential methodology, as is typical for adaptive designs, is that it is most effective in practice when outcome accruement following inclusion in the experiment is fast relative to the entire length of the study (i.e., the enrollment rate), as we make the underlying assumption that outcome accruement completes between interim analyses, removing potential issues caused by delayed outcomes. That is, the theoretical efficiency gains are most likely to be realized in reality in this case. This is not to say, however, that a group-sequential approach cannot be useful when the study duration is short, as accruement could be paused in each stage when the required sample size has been achieved. This though would likely come at a cost to the overall time taken to complete the experiment. Thus, the utility of a group-sequential approach may often depend on the willingness to trade an increased study length for a smaller required sample size.

Moreover, we here assume that all observations are accrued following a fixed and common period of observation. This may make application in fields such as manufacturing easier than many clinical research settings where observation time may be more difficult to standardize in this manner. For scenarios with highly variable observation times, alternative methodology for group sequential design (e.g., Xia and Hoover 2007) would likely be more appropriate. At the design stage of an experiment, however, provided only small variability in the observation times is anticipated, our methods could provide a simple means of determining the approximately required sample size.

We specified our null hypothesis in a composite form, and also powered the trial across a range of possible mean event rates. We made these choices as in many settings, in particular in clinical research, it would likely difficult to nominate single points at which to control the error-rates. This came at a cost, however, in that a method to control the maximal error-rates across the sets Λ_0_ and Λ_1_ was required. Whilst this was readily achieved, at least theoretically, for the normal approximation design, this was not the case for the exact approach. We thus retained one-dimensional searches for maximal stopping probabilities in our specification of the *a_k_* and *r_k_*. It is important to acknowledge that this approach may in general lead to conservative designs as, for example, the value of *λ* ∈ Λ_0_ that maximizes *R_k_*(·) may not be equal across *k* ∈ {1, …, *K*}. In practice, this appears to not be the case, as we were able to identify highly efficient designs with maximal error-rates close to their desired level. Nonetheless, one possible solution to reducing this conservatism could be to conduct a test conditional on the number of observed events, similar to the approaches of Xia and Hoover (2007) and Grayling et al. (2017). This remains as a possible avenue for future research.

We finish with a discussion of an important point: the inherent pros and cons of the two described design determination procedures. The exact procedure is, as discussed, preferable precisely because it is exact. That is, it guarantees control of the type-I and type-II error-rates. In contrast, the normal approximation approach may not provide such error-rate control in practice, and verifying that it does via simulation may be particularly time consuming. However, our presented simulation study does suggest that in many settings the error-rates of normal approximation designs may often be close to their nominal levels. Furthermore, from Table 1 we observe that in certain cases, for a given ***w***, the normal approximation designs have lower ESSs than the corresponding exact designs. This is a consequence of the fact that ***a***, ***r*** ∈ ℝ^*K*^, rather than ℤ^*K*^ for the normal approximation design, and so can be more precisely tailored to each possible value of *n*. In addition, provided additional simulations are not required to confirm the theoretical operating characteristics, the optimal normal approximation designs are typically faster to identify than their exact counterparts. This may lead us to conclude that the normal approximation approach may be preferred when only approximate error-rate control is desired, or when a search for an exact design would be time consuming. In contrast, if one wishes to be certain that the error-rates are controlled, or wishes to know for sure that the correct optimal or near-optimal design has been chosen, the exact approach may be preferred. Finally, in particular, whilst there does not appear to be a simple rule through which we can specify the estimated error-rates of a normal approximation design will begin to deviate from their empirical values, similar to design for Bernoulli outcome data, utilization of an exact approach would be most expedient when the requisite sample size is small (i.e., when the anticipated mean response is small and/or *δ* is large). Heeding this advice, group sequential tests for Poisson distributed outcome variables can then be determined which substantially reduce the requisite sample size compared to single-stage approaches.

## Supplementary Material

Supplemental data for this article can be accessed here.

Supplementary file

## Figures and Tables

**Table 1:** The optimal two and three-stage designs for Example 1 are shown, based on the exact (optimal and near-optimal) and normal approximation approaches. For comparison, the single-stage designs are also given. Note that for brevity we write *α′* = *α′*(*n*, ***a***, ***r***), *β′* = *β′*(*n*, ***a***, ***r***), *ESS*(*λ*_ESS_, * λ*ESS) = *ESS*(*λ*_ESS_, *λ*_ESS_ | *n*, ***a***, ***r***), and similarly for *ESS*(*λ*_ESS_, *λ*_ESS_–*δ*). The type-I error-rates and power figures are given to 3 dp, whilst ESSs are given to 1 dp.

*K*	*w*	*π_A_*	*π_R_*	*n*	*a*	*r*	*α*′	1–*β*′	*ESS*(*λ*_ESS,_ *λ*_ESS_)	*ESS*(*λ*_ESS,_ *λ*_ESS_–δ)	*2Kn*
Exact: Optimal											
1	N/A	N/A	N/A	73	110	110	0.049	0.800	146.0	146.0	146
2	***w***_1_	N/A	N/A	40	(35, 108)	(141, 108)	0.050	0.801	92.8	151.0	160
2	***w***_2_	N/A	N/A	42	(–26,138)	(92, 138)	0.050	0.800	148.5	123.1	168
2	***w***_3_	N/A	N/A	43	(38, 126)	(98, 126)	0.050	0.800	98.5	126.1	172
2	***w***_4_	N/A	N/A	38	(22, 109)	(127, 109)	0.050	0.800	95.9	142.3	152
2	***w***_5_	N/A	N/A	38	(–52,118)	(101, 118)	0.050	0.800	147.3	127.5	152
2	***w***_6_	N/A	N/A	39	(25, 113)	(109, 113)	0.050	0.800	96.4	133.3	156
Exact: Near-optimal											
1	N/A	0.2	0.05	73	110	110	0.049	0.800	146.0	146.0	146
2	***w***_1_	(0.14,0.06)	(0.01,0.04)	42	(41, 112)	(118, 112)	0.049	0.802	94.6	142.2	168
2	***w***_2_	(0.02,0.18)	(0.03,0.02)	41	(–8,132)	(94, 132)	0.049	0.800	130.5	124.1	164
2	***w***_3_	(0.12,0.08)	(0.03,0.02)	44	(40, 130)	(98, 130)	0.049	0.800	99.9	126.6	176
2	***w***_4_	(0.08,0.12)	(0.005,0.045)	38	(20, 110)	(124, 110)	0.049	0.800	97.4	141.2	152
2	***w***_5_	(0.04,0.16)	(0.02,0.03)	39	(5, 120)	(100, 120)	0.050	0.802	112.8	127.5	156
2	***w***_6_	(0.1,0.1)	(0.015,0.035)	40	(28, 116)	(107, 116)	0.049	0.801	97.0	132.8	160
3	***w***_1_	(0.12,0.03,0.05)	(0.01,0.015,0.025)	30	(19, 49, 121)	(100, 125, 121)	0.049	0.800	81.7	129.9	180
3	***w***_2_	(0.03,0.06,0.11)	(0.015,0.02,0.015)	28	(–13,45,133)	(90, 113, 133)	0.049	0.800	101.4	121.3	168
3	***w***_3_	(0.12,0.03,0.05)	(0.02,0.02,0.01)	33	(23, 59, 144)	(92, 119, 144)	0.049	0.803	86.0	122.9	198
3	***w***_4_, ***w***_6_	(0.06,0.06,0.08)	(0.01,0.01,0.03)	27	(–1,47,117)	(95, 127, 117)	0.049	0.801	88.4	129.1	162
3	***w***_5_	(0.03,0.06,0.11)	(0.01,0.02,0.02)	27	(–14,42,125)	(95, 113, 125)	0.049	0.801	99.4	122.7	162
Normal approximation											
1	N/A	0.2	0.05	71	1.64	1.64	0.050	0.802	142.0	142.0	142
2	***w***_1_	(0.12,0.08)	(0.005,0.045)	39	(0.67,1.57)	(2.58,1.57)	0.050	0.801	97.1	112.4	156
2	***w***_2_	(0.04,0.16)	(0.03,0.02)	40	(0.12,1.86)	(1.88,1.86)	0.050	0.802	113.7	96.1	160
2	***w***_3_	(0.1,0.1)	(0.015,0.035)	39	(0.57,1.65)	(2.17,1.65)	0.050	0.802	99.1	100.7	156
2	***w***_4_	(0.08,0.12)	(0.005,0.045)	37	(0.40,1.62)	(2.58,1.62)	0.050	0.800	99.2	109.3	148
2	***w***_5_	(0.04,0.16)	(0.015,0.035)	37	(0.05,1.70)	(2.17,1.70)	0.050	0.801	108.4	98.3	148
2	***w***_6_	(0.08,0.12)	(0.015,0.035)	38	(0.42,1.67)	(2.17,1.67)	0.050	0.801	100.5	99.4	152
3	***w***_1_	(0.12,0.03,0.05)	(0.01,0.01,0.03)	29	(0.42,0.80,1.60)	(2.33,2.21,1.60)	0.050	0.802	85.2	92.3	174
3	***w***_2_	(0.03,0.03,0.14)	(0.025,0.01,0.015)	28	(–0.31,0.50,1.92)	(1.96,2.13,1.92)	0.050	0.803	103.5	83.6	168
3	***w***_3_	(0.09,0.06,0.05)	(0.02,0.015,0.015)	30	(0.28,1.06,1.78)	(2.05,2.00,1.78)	0.050	0.800	87.8	86.0	180
3	***w***_4_, ***w***_6_	(0.06,0.03,0.11)	(0.01,0.01,0.03)	26	(–0.05,0.52,1.69)	(2.33,2.22,1.69)	0.050	0.802	90.9	89.0	156
3	***w***_5_	(0.03,0.03,0.14)	(0.015,0.01,0.025)	26	(–0.37,0.42,1.77)	(2.17,2.19,1.77)	0.050	0.803	99.7	85.9	156
