# Supplementary material for: Exact group sequential designs for two-arm experiments with Poisson distributed outcome variables
Source: Commun Stat Theory Methods. Author manuscript; Available in PMC 2021 Jan 5. (PMC7116540; doi:10.1080/03610926.2019.1628273)
Supplement: Supplementary file [file EMS84073-supplement-Supplementary_file.pdf]

Supplementary Material for 'Exact group sequential designs for two-arm experiments with Poisson distributed outcome variables'

1. Maximal type-I and type-II error-rates for composite null hypotheses

Here, we expand on the issues associated with controlling the error-rates of an exact group-sequential trial specified in terms of the sets Λ_0 and Λ_1. As noted in the main manuscript, such a specification is in general a useful one as in many design scenarios of practical interest it may be difficult, or in fact impossible, to nominate a point null (or indeed alternative) hypothesis of the form $H_0 : \lambda_1 = \lambda_2 = \lambda_0 \in \mathbb{R}^+$.

First, it is useful to recognise that the type-I and type-II error-rates can vary widely across the sets Λ_0 and Λ_1, and thus it would not be appropriate to use an approach similar to that employed for the normal approximation design, and work in the design search in terms of the error-rates calculated at singular points. To this end, Supplementary Figure 1 presents the type-I and type-II error-rates of the exact group sequential design with $n = 42$, $\boldsymbol{a} = (41, 112)$, and $\boldsymbol{r} = (118, 112)$, across $\lambda_1 \in \Lambda_0 = \Lambda_1 = [15, 30]$ (the near-optimal design for $\boldsymbol{w}_1$ in Example 1). As noted, their minimal and maximal values differ substantially.

However, more importantly, we may hope that we could identify analytically where the maxima occurs. In particular, may hope that it will always occur on the boundary (i.e., the infima or suprema) of the sets Λ_0 and Λ_1. As we discussed in the main manuscript,

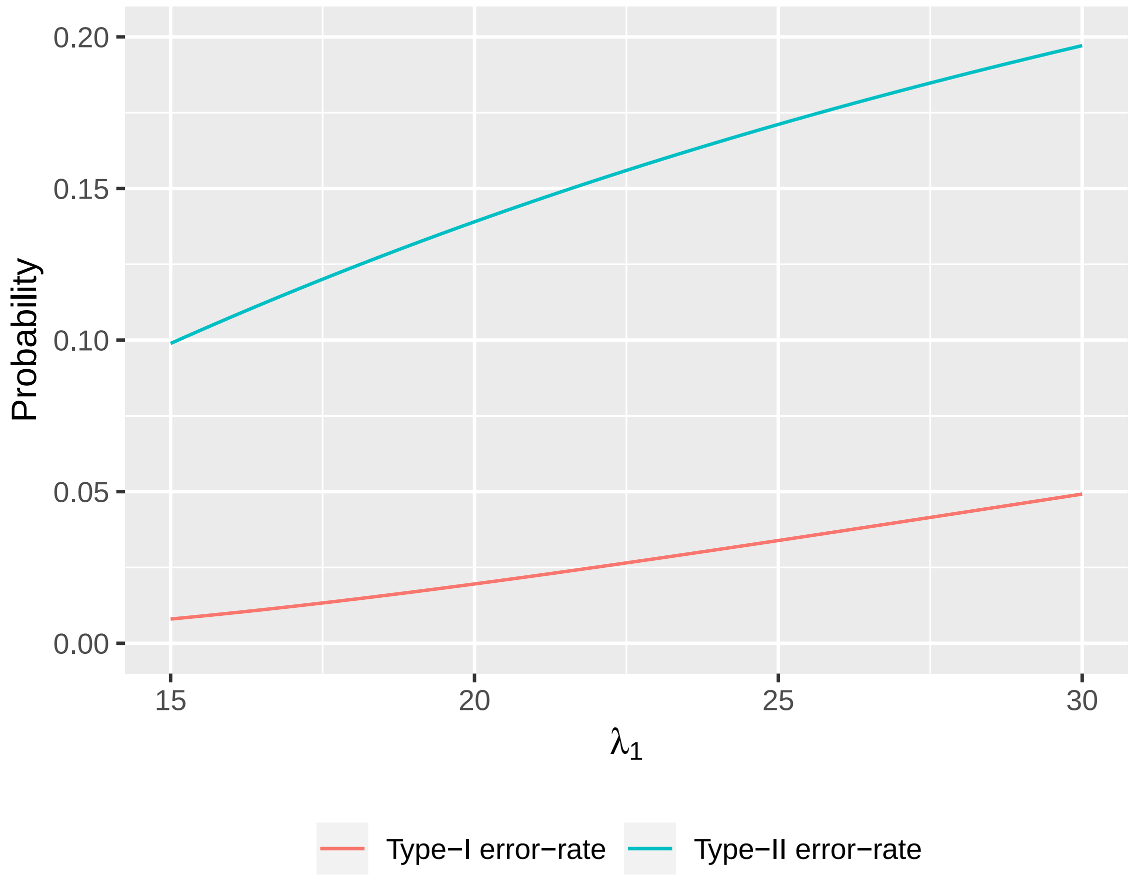

Supplementary Figure 1: Type-I ($\lambda_1 = \lambda_2$) and type-II ($\lambda_1 = \lambda_2 + \delta = \lambda_2 + 2.25$) error-rates of the exact design with $n = 42$, $\boldsymbol{a} = (41, 112)$, and $\boldsymbol{r} = (118, 112)$, when $\lambda_1 \in \Lambda_0 = \Lambda_1 = [15, 30]$.

proving this would likely be extremely challenging, whilst for the type-I error-rate at least, the location of the maxima does not appear to have a simple form. To demonstrate this, we performed a search for all 1,410,780 with $n \in \{1, 2, \dots, 20\}$ and $a_1, r_1, r_2 \in \{-2n, -2n + 1, \dots, 2n\}$, $a_1 < r_1 - 1$, to identify the location of the maximal type-I error-rate across $\lambda_1 = \lambda_2 \in [15, 30]$. We found that for 704,492 of the designs the type-I error-rate was maximised at $\lambda_1 = \lambda_2 = 15$, for 689,951 it was maximised at $\lambda_1 = \lambda_2 = 30$, and for 16,337 it was maximised at some $\lambda_1 = \lambda_2 \in (15, 30)$. Whilst this was not the case for the type-II error-rate, which appeared to be consistently maximised at $\lambda_1 = \lambda_2 + \delta = \sup(\Lambda_1)$. Nonetheless, without a formal proof of where the error-rates will be maximised, we retain a search for the maximal stopping probabilities in our near-optimal design determination procedure. Note that code to replicate our results is available at https://github.com/mjg211/article_code.

2. Example 2

Here, to further explore the utility of our group sequential designs, we reconsider the example from Menon et al. (2011). They considered a clinical research scenario in which investigators are interested in the relapse rate per year of an infectious disease on two treatments. The hypothesised rate for the new treatment ($j = 2$) was 5.15, whilst the standard treatment ($j = 1$) was expected to have rate 6.25, giving $\Lambda_0 = \Lambda_1 = \lambda_0 = 6.25$ and $\delta = 1.1$ within the notation of the main manuscript. Finally, they desired 90% power for a type-I error-rate of 5%. Accordingly we take $\beta = 0.1$ and $\alpha = 0.05$, and as for Example 1 we consider the optimal designs for

$$\boldsymbol{w}_1 = (1, 0, 0),\ \boldsymbol{w}_2 = (0, 1, 0),\ \boldsymbol{w}_3 = (1/2, 1/2, 0),$$
$$\boldsymbol{w}_4 = (1/2, 0, 1/2),\ \boldsymbol{w}_5 = (0, 1/2, 1/2),\ \boldsymbol{w}_6 = (1/3, 0, 1/3),$$

when $K \in \{2, 3\}$.

For the case $K = 2$ we consider all combinations of $\boldsymbol{\pi}_A$ and $\boldsymbol{\pi}_R$, conforming to our requirements from earlier, with $(\pi_{A1}, \pi_{R1}) \in \{0.01, 0.02, \dots, 0.08, 0.09\} \times \{0.005, 0.01, \dots, 0.045\}$. Whilst for $K = 3$ we examine all permissible combinations with $(\pi_{A1}, \pi_{A2}, \pi_{R1}, \pi_{R2}) \in \{0.01, 0.03, 0.05, 0.07\}^2 \times \{0.01, 0.015, \dots, 0.035\}^2$.

Thus the optimal designs for $K \in \{2, 3\}$, amongst the considered $\boldsymbol{\pi}_A$ and $\boldsymbol{\pi}_R$, were determined for the six stated values of $\boldsymbol{w}$. They are displayed, along with the corresponding single-stage designs based on the exact and normal approximation methods, in Supplementary Table 1.

We observe that, as for Example 1, utilising a group sequential approach reduces the ESS when $\lambda_1 = \lambda_2 = \lambda_0$ and when $\lambda_1 = \lambda_2 + \delta = \lambda_0$ relative to using a single-stage design. The ESS when $\lambda_1 = \lambda_2 = \lambda_0$ can be reduced by as much as 38% and 40% using the normal approximation or exact approach respectively (for $K = 3$, compared to their respective required sample sizes when $K = 1$). Similarly, as in Example 1, increasing the value of K allows us to increase efficiency further in terms of the ESS, at a cost to

the maximal possible sample sizes. Finally, once more, for both design approaches, there exist designs that require only minor increases to the maximal possible sample size, which bring sizeable reductions to the ESSs.

Supplementary Table 1: The optimal two and three-stage designs for Example 2 are shown, amongst the considered $\boldsymbol{\pi}_A$ and $\boldsymbol{\pi}_R$, based on the exact and normal approximation approaches. For comparison, the single-stage designs are also given. Note that for brevity we write $\alpha' = \alpha'(\lambda_0, n, \boldsymbol{a}, \boldsymbol{r})$, $\beta' = \beta'(\lambda_0, n, \boldsymbol{a}, \boldsymbol{r})$, $ESS(\lambda_0, \lambda_0) = ESS(\lambda_0, \lambda_0 \mid n, \boldsymbol{a}, \boldsymbol{r})$, and similarly for $ESS(\lambda_0, \lambda_0 - \delta)$. The type-I error-rates and power figures are given to 3 dp, whilst ESSs are given to 1 dp.

K	$\boldsymbol{w}$	$\boldsymbol{\pi}_A$	$\boldsymbol{\pi}_R$	n	$\boldsymbol{a}$	$\boldsymbol{r}$	α'	$1-\beta'$	$ESS(\lambda_0,\lambda_0)$	$ESS(\lambda_0,\lambda_0-\delta)$	$2Kn$
					Exact: Near-optimal						
1	N/A	0.1	0.05	87	55	55	0.049	0.905	174.0	174.0	174
2	$\boldsymbol{w}_1$	$(0.06, 0.04)$	$(0.005, 0.045)$	47	$(16, 55)$	$(63, 55)$	0.049	0.901	118.1	152.4	188
2	$\boldsymbol{w}_2$	$(0.04, 0.06)$	$(0.025, 0.025)$	48	$(12, 62)$	$(49, 62)$	0.048	0.900	124.4	133.3	192
2	$\boldsymbol{w}_3$	$(0.06, 0.04)$	$(0.025, 0.025)$	50	$(18, 62)$	$(50, 62)$	0.048	0.900	121.8	135.1	200
2	$\boldsymbol{w}_4$	$(0.03, 0.07)$	$(0.005, 0.045)$	44	$(6, 55)$	$(61, 55)$	0.049	0.900	123.4	147.7	176
2	$\boldsymbol{w}_5$, $\boldsymbol{w}_6$	$(0.02, 0.08)$	$(0.015, 0.035)$	45	$(3, 58)$	$(52, 58)$	0.049	0.901	129.9	136.5	180
3	$\boldsymbol{w}_1$	$(0.05, 0.03, 0.02)$	$(0.01, 0.015, 0.025)$	35	$(6, 31, 60)$	$(50, 62, 60)$	0.048	0.901	104.6	130.7	210
3	$\boldsymbol{w}_2$	$(0.03, 0.03, 0.04)$	$(0.03, 0.01, 0.01)$	37	$(2, 32, 75)$	$(41, 65, 75)$	0.049	0.902	114.4	122.4	222
3	$\boldsymbol{w}_3$	$(0.05, 0.03, 0.02)$	$(0.02, 0.015, 0.015)$	37	$(7, 34, 67)$	$(45, 62, 67)$	0.048	0.902	107.2	124.5	222
3	$\boldsymbol{w}_4$, $\boldsymbol{w}_6$	$(0.03, 0.03, 0.04)$	$(0.01, 0.01, 0.03)$	32	$(-1, 24, 59)$	$(48, 64, 59)$	0.047	0.901	108.1	130.5	192
3	$\boldsymbol{w}_5$	$(0.01, 0.01, 0.08)$	$(0.01, 0.015, 0.025)$	31	$(-10, 9, 61)$	$(48, 64, 59)$	0.048	0.903	125.8	127.9	186
					Normal approximation						
1	N/A	0.1	0.05	81	1.64	1.64	0.050	0.901	162.0	162.0	162
2	$\boldsymbol{w}_1$	$(0.06, 0.04)$	$(0.005, 0.045)$	45	$(0.63, 1.58)$	$(2.58, 1.58)$	0.050	0.902	113.3	143.3	180
2	$\boldsymbol{w}_2$	$(0.02, 0.08)$	$(0.025, 0.025)$	44	$(0.11, 1.79)$	$(1.96, 1.79)$	0.050	0.900	126.0	123.2	176
2	$\boldsymbol{w}_3$	$(0.06, 0.04)$	$(0.025, 0.025)$	48	$(0.70, 1.72)$	$(1.96, 1.72)$	0.050	0.903	116.8	127.0	192
2	$\boldsymbol{w}_4$	$(0.03, 0.07)$	$(0.005, 0.045)$	42	$(0.23, 1.63)$	$(2.58, 1.63)$	0.050	0.902	117.9	138.5	168
2	$\boldsymbol{w}_5$	$(0.01, 0.09)$	$(0.015, 0.035)$	42	$(-0.22, 1.71)$	$(2.17, 1.71)$	0.050	0.901	131.9	127.1	168
2	$\boldsymbol{w}_6$	$(0.03, 0.07)$	$(0.015, 0.035)$	43	$(0.26, 1.69)$	$(2.17, 1.69)$	0.050	0.901	119.0	127.6	172
3	$\boldsymbol{w}_1$	$(0.05, 0.03, 0.02)$	$(0.01, 0.015, 0.025)$	33	$(0.23, 1.00, 1.63)$	$(2.33, 2.07, 1.63)$	0.050	0.900	99.9	120.0	198
3	$\boldsymbol{w}_2$	$(0.03, 0.03, 0.04)$	$(0.025, 0.015, 0.01)$	34	$(0.02, 0.98, 1.96)$	$(1.96, 1.99, 1.96)$	0.050	0.901	107.5	112.5	204
3	$\boldsymbol{w}_3$	$(0.05, 0.03, 0.02)$	$(0.02, 0.015, 0.015)$	35	$(0.28, 1.09, 1.77)$	$(2.05, 2.00, 1.77)$	0.050	0.901	102.0	115.0	210
3	$\boldsymbol{w}_4$, $\boldsymbol{w}_6$	$(0.01, 0.03, 0.06)$	$(0.01, 0.01, 0.03)$	29	$(-0.57, 0.68, 1.71)$	$(2.33, 2.22, 1.71)$	0.050	0.902	111.9	118.4	174
3	$\boldsymbol{w}_5$	$(0.01, 0.01, 0.08)$	$(0.01, 0.015, 0.025)$	29	$(-0.57, 0.26, 1.76)$	$(2.33, 2.08, 1.76)$	0.050	0.902	119.2	116.8	174
